# Machine learning algorithms to predict atypical metastasis of colorectal cancer patients after surgical resection

**DOI:** 10.3389/fsurg.2022.1049933

**Published:** 2023-01-06

**Authors:** Xiaoyan Yang, Wei Yu, Feimin Yang, Xiujun Cai

**Affiliations:** ^1^ Department of General Surgery, Key Laboratory of Endoscopic Technique Research of Zhejiang Province, Sir Run Run Shaw Hospital, School of Medicine, Zhejiang University, Hangzhou, China; ^2^ Department of Surgical Oncology, Sir Run Run Shaw Hospital, School of Medicine, Zhejiang University, Hangzhou, China

**Keywords:** atypical metastasis, machine learning, predictive model, colorectal cancer, surgery

## Abstract

**Background:**

The prognosis of colorectal cancer with atypical metastasis is poor. However, atypical metastasis was less common and under-appreciated.

**Methods:**

In this study we attempted to present the first machine learning models to predict the risk of atypical metastasis in colorectal cancer patients. We evaluated the differences between metastasis and non-metastasis groups, assessed factors associated with atypical metastasis using univariate and multivariate logistic regression analyses, and preliminarily developed the multiple machine learning models to predict atypical metastasis.

**Results:**

168 patients were included. Prognostic Nutritional Index (PNI) [OR =  0.998; *P* = 0.030], Cancer antigen 19–9 (CA19-9) [OR = 1.011; *P* = 0.043] and MR-Distance [-mid OR = 0.289; *P* = 0.009] [-high OR = 0.248; *P* = 0.021] were shown to be independent risk factors for the atypical metastasis *via* multivariate analysis. Furthermore, the machine learning model based on AdaBoost algorithm (AUC: 0736) has better predictive performance comparing to Logistic Regression (AUC: 0.671) and KNeighbors Classifier (AUC: 0.618) by area under the curve (AUC) in the validation cohorts. The accuracy, sensitivity, and specificity of the model trained using the Adaboost method in the validation set are 0.786, 0.776 and 0.700, while 0.601, 0.933, 0.508 using Logistic Regression and 0.743, 0.390, 0.831 using KNeighbors Classifier.

**Conclusion:**

Machine-learning approaches containing PNI, CA19-9 and MR-Distance show great potentials in atypical metastasis prediction.

## Introduction

Colorectal cancer (CRC) is common worldwide and associated with significant mortality (1). Distant metastasis is a major contributor to the mortality of colorectal cancer patients, approximately 50% of patients develop metastases despite surgical resection (2). The main sites of metastases were liver, lung, peritoneum and peripheral lymph nodes. Atypical metastasis of colorectal cancer refers to other uncommon metastatic sites, including bone, adrenal gland, ovary, brain, pancreas and spleen (3, 4). Atypical metastasis commonly represents a manifestation of advanced colorectal cancer, which correlates with a poor survival prognosis (5).

Atypical metastasis diagnoses were confirmed by clinical manifestations and radiographic imaging. The clinical symptoms of atypical metastasis are determined by the specific target organ. Bone metastasis patients often suffer from tumor-induced bone pain, pathological bone fractures, and spinal cord compression (6); Brain metastases patients may present with headache, dizziness and seizures (7). However, given the low incidence of atypical metastasis in colorectal cancer, high-cost imaging test at follow-up is not clinically recommended until specific symptoms are present. Indeed, due to the low incidence rates and low detection rates, atypical metastasis has been consistently neglected in clinical practice and research.

To date, studies related to atypical metastasis of colorectal cancer are mainly limited to case reports (8, 9). To our knowledge, there are currently no objective tools that identify colorectal cancer patients at risk for atypical metastasis. The purpose of this study is to identify the risk factors for atypical metastasis in colorectal cancer patients after surgical resection and first explore the application of machine learning toward providing potential clinical predictive tools for atypical metastasis.

## Methods

### Patients

This study was approved by the institutional review board of Sir Run Run Shaw hospital, and the requirement for patient consent waived. Atypical metastasis of colorectal cancer in this study refers to other metastases excluding local direct infiltration and distant metastases in liver metastases and lung metastases. Consecutive patients were identified that fulfilled all inclusion and none of the exclusion criteria from January 2010 to December 2018. Inclusion criteria were as follows: (1) patients with rectal cancer confirmed by surgical pathology; (2) without a history of comorbid other malignancies; and (3) complete medical record and follow-up information. Exclusion criteria were as follows: (1) patients with synchronous metastasis of rectal cancer; (2) incomplete medical history or follow-up lost; and (3) undergone radiotherapy, chemotherapy or neoadjuvant radiotherapy.

Preoperative variables including the *α*-fetoprotein (AFP), C-reactive protein (CRP), Cancer antigen 19-9 (CA19-9), albumin, aspartate transaminase (AST), alanine aminotransferase (ALT) and lymphocyte count level were measured. Prognostic Nutritional Index (PNI) was defined as 10 × serum albumin (g/L) + 0.005 × lymphocyte count (per mm3). Besides, MRI-based imaging measures, including the were also evaluated. The definition of MR-distance was tumor distance from the anal verge confirmed from preoperative magnetic resonance (MR) images.

### MRI protocol and image analysis

MRI was performed with a 3.0 T MR scanner (Signa HDxt, GE Healthcare, Chicago, IL, USA). Imaging sequences included routine high-resolution axial T2-weighted imaging (HRT2WI) and DWI. Image acquisition parameters for HRT2WI were as follows: fast recovery fast spin echo, repetition time = 3,300 ms, echo time = 130 ms, slice thickness = 3.0 mm, gap = 3.0 mm, matrix = 512 × 512, echo train length = 20, and field of view = 160 × 160 mm. The parameters for DWI was as follows: b = 800s/mm2, repetition time = 5,900 ms, echo time = 66 ms, slice thickness = 5.0 mm, matrix = 256 × 256, field of view = 290 × 290 mm, and gap = 6.0 mm.

All images were reviewed on ITK-SNAP software using the picture archiving and communication system. Regions of interest were measured on the HRT2WI and DWI sequence. The image analysis was conducted by an experienced radiologist with 10 years of experience, who was blinded to the clinical information.

### Statistical analysis

The machine learning analyses were performed using scikit-learn version 0.22.1 in Python 3.7. The R statistical package version 3.6.3 was used, with libraries “mstate”, “pROC”, “rms”, “mstate” and “dplyr”. Differences between metastasis and non-metastasis groups were analyzed using *t*-test, Mann-Whitney *U* test or Chi-square test. Univariate and multivariate logistic regression analyses were utilized to identify factors associated with atypical metastasis of colorectal cancer. Comparison between AdaBoost (learning rate: 1.0, estimators: 50), Logistic Regression (C: 1.0, max_iter: 100, penalty: l2) and KNeighbors classifier (neighbors: 5, weights: uniform) based on PNI, CA19-9 and MR-Distance were assessed by AUC, as well as sensitivity, specificity and accuracy. AUC mean (and standard deviation) determined by 5-fold cross-validation. Differences were considered statistically significant at *P* value < 0.05.

## Results

168 patients were included in this study. Overall, 31 patients were diagnosed with atypical metastasis during follow-up, whereas the remaining 137 patients presented no metastasis at any sites. Among them, bone metastases were identified in 23 patients, while adrenal metastases in 3 patients, splenic metastases in 2 patients, and one patient each for ovarian, brain, and skeletal muscle metastases.

**Figure 1:**
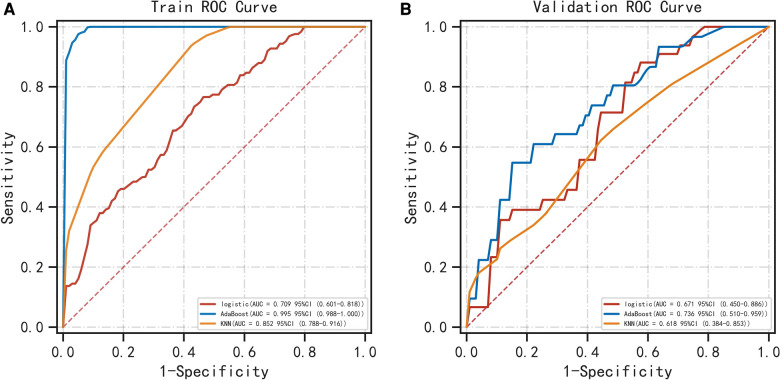
Comparison of ROC curves for prediction of atypical metastasis of colorectal cancer. The three ROC curves separately shown are for the logistic regression, AdaBoost algorithm and KNN classifier in the training (**A**) and validation (**B**) cohorts. ROC, receiver operating characteristic. KNN classifier, KNeighbors classifier.

Table 1 shows the demographic data. Table 2 shows that PNI [OR =  0.998; *P* = 0.030], CA19-9 [OR = 1.011; *P* = 0.043] and MR-Distance [-mid OR = 0.289; *P* = 0.009] [-high OR = 0.248; *P* = 0.021] were independent risk factors for atypical metastasis. Furthermore, Figure 1 shows that the preliminary machine learning model based on AdaBoost algorithm has better predictive performance comparing to Logistic Regression and KNeighbors Classifier in the training (AUC: 0.995, 0.709, 0.852 respectively) and validation cohorts (AUC: 0736, 0.671, 0.618 respectively). As shown in Table 3, the accuracy, sensitivity, and specificity of the model trained using the Adaboost method in the validation set are 0.786, 0.776 and 0.700, while 0.601, 0.933, 0.508 using Logistic Regression and 0.743, 0.390, 0.831 using KNeighbors Classifier.

**Table 1:** Patient clinical characteristics.

Characteristic	Non-metastasis (*n* = 137)	metastasis (*n* = 31)	*P*
Age	64.599 (10.483)	62.516 (12.234)	0.338
Gender (male)	75 (54.745)	16 (51.613)	0.752
BMI	22.945 (3.368)	22.848 (3.342)	0.889
Total protein	66.707 (7.414)	65.596 (5.653)	0.437
ALP	72.159 (17.618)	71.040 (12.116)	0.678
Albumin	39.794 (4.382)	39.059 (3.486)	0.386
CRP	2.650[0.900,9.800]	2.400[1.350,6.700]	0.800
LDH	183.000[164.667,203.000]	178.667[162.000,195.500]	0.458
CA125	9.600[7.500,12.690]	9.570[8.535,12.210]	0.510
CEA	3.430[2.050,6.200]	4.010[3.065,8.220]	0.122
CA199	11.570[7.765,18.400]	12.635[7.290,18.380]	0.596
AFP	2.690[1.960,3.480]	3.003[2.330,3.930]	0.142
CA153	10.680[8.651,13.220]	9.800[8.667,12.760]	0.563
PNI	373.008[0.012,421.009]	191.508[0.009,375.008]	0.014
**MR-Distance**			
Low	28 (20.438)	15 (48.387)	0.006
Mid	73 (53.285)	11 (35.484)	
High	36 (26.277)	5 (16.129)	
MR-EMVI (present)	49 (35.766)	13 (41.935)	0.520

ALP, alkaline phosphatase; CRP, C-reactive protein; LDH, lactate dehydrogenase; CEA, carcinoembryonic antigen; CA125, CA199, CA153 carbohydrate antigen (CA) 125, CA199, CA153; AFP, alpha-fetoprotein; PNI, prognostic nutritional index. MR-Distance distance from anal verge from magnetic resonance image. MR-EMVI Extramural vascular invasion from magnetic resonance image.

^#^ Gender, MR-Distance and MR-EMVI were analyzed using Chi-square test and expressed as *n* (%). Age, BMI, Total protein, ALP and Albumin were analyzed using *t*-test and expressed as mean (SD). CRP, LDH, CA125, CEA, CA199, AFP, CA153 and PNI were analyzed using Mann-Whitney *U* test and expressed as median (range).

**Table 2:** Univariate and multivariate analysis of prognostic factors of atypical metastasis in colorectal cancer patients

Variables	Univariate			Multivariate		
	Odds ratio	95% confidence interval	*P*	Odds ratio	95% confidence interval	*P*
Age	0.983	[0.948,1.018]	0.336			
Gender (male)	0.882	[0.404,1.925]	0.752			
Total protein (g/L)	0.978	[0.926,1.033]	0.434			
ALP (U/L)	0.996	[0.973,1.020]	0.737			
Albumin (g/L)	0.960	[0.877,1.052]	0.384			
CRP (mg/L)	0.995	[0.978,1.011]	0.537			
LDH (IU/L)	0.994	[0.981,1.008]	0.423			
CA125 (U/ml)	0.997	[0.948,1.049]	0.909			
CEA (ng/ml)	1.017	[0.994,1.040]	0.141			
CA199 (IU/ml)	1.010	[1.000,1.021]	**0** **.** **046**	1.011	[1.000,1.021]	**0** **.** **043**
AFP (ng/ml)	1.128	[0.880,1.446]	0.341			
CA153 (U/L)	0.967	[0.870,1.076]	0.542			
PNI	0.998	[0.996,1.000]	**0** **.** **024**	0.998	[0.996,1.000]	**0** **.** **030**
**MR-Distance**						
Low						
Mid	0.281	[0.115,0.686]	**0** **.** **005**	0.289	[0.114,0.735]	**0** **.** **009**
High	0.259	[0.084,0.800]	**0** **.** **019**	0.248	[0.076,0.810]	**0** **.** **021**
MR-EMVI	1.297	[0.586,2.870]	0.521			

ALP, alkaline phosphatase; CRP, C-reactive protein; LDH, lactate dehydrogenase; CEA, carcinoembryonic antigen; CA125, CA199, CA153 carbohydrate antigen (CA) 125, CA199, CA153; AFP, alpha-fetoprotein; PNI, Ppognostic nutritional index. MR-Distance distance from anal verge from magnetic resonance image. MR-EMVI Extramural vascular invasion from magnetic resonance image.

**Table 3:** Predictive performance of three models for predicting atypical metastasis of colorectal cancer patients.

Model	Accuracy	Sensitivity	Specificity
**Training cohort**			
Logistic Regression	0.656 (0.539–0.774)	0.724 (0.565–0.883)	0.648 (0.468–0.827)
KNN	0.833 (0.816–0.850)	0.920 (0.763–1.077)	0.604 (0.453–0.755)
AdaBoost	0.954 (0.941–0.967)	0.992 (0.976–1.008)	0.949 (0.928–0.970)
**Validation cohort**			
Logistic Regression	0.601 (0.469–0.733)	0.933 (0.803–1.064)	0.508 (0.289–0.728)
KNN	0.743 (0.677–0.810)	0.390 (0.219–0.562)	0.831 (0.692–0.971)
AdaBoost	0.786 (0.738–0.833)	0.776 (0.651–0.901)	0.700 (0.512–0.888)

## Discussion

The main finding of the current study is that Prognostic Nutritional Index (PNI), Cancer antigen 19-9 (CA19-9) and MR-Distance were independent risk factors for the atypical metastasis, and the machine learning model based on AdaBoost algorithm may have good predictive performance. The results support that machine learning tools offer the promise of being more generalizable and applicable to predicting atypical metastasis in colorectal cancer after resection.

In our study, PNI and serum CA19-9 have been shown to be an independent risk factor for predicting atypical metastasis in colorectal cancer. Nozoe et al. first revealed that PNI less than 40 was independently correlated with a worse prognosis of patients with colorectal cancer (10). Subsequently, Mohri et al. clarified that relatively low levels of PNI, which is in a significantly malnourished state, can more accurately reflect the nutritional status of colorectal cancer patients (11). Schwegler et al. (12) and Kwag et al. (13) respectively found that nutritional risk is an independent risk factor for postoperative morbidity in colorectal cancer, which is consistent with the results of our study. Several studies have further demonstrated the predictive value of PNI for overall survival and tumor-free survival in patients with colorectal carcinoma (14–16). However, studies of PNI in the risk of colorectal cancer metastasis are rare and have not been explored especially in atypical metastases. CA19-9 have proven serve as the indicators for the predictive outcome in multiple types of cancer, although the specificity is unsatisfied (17–20). A study by Yang proposed that preoperative serum CA19-9 could be one of the independent prognostic factors in determining 5-year tumor-free survival in colorectal cancer (21). Subsequent findings suggest a significant association between CA19-9 and poor prognosis of colorectal cancer. In our study, low PNI and high CA19-9 are associated with the risk of atypical metastasis in colorectal cancer.

Artificial intelligence and machine learning approaches are increasingly applied in medicine (22, 23). Our results in the validation set suggest that the overall effectiveness of traditional logistic regression method in assessing the risk of atypical metastasis is not good (AUC = 0.671), relatively, the comprehensive performance of Adaboost algorithm is better (AUC = 0.736). However, the sensitivity of logistic regression model is the highest as seen in the validation set, indicating such classical analysis also has its advantages.

The advantage of this study is first evaluation the machine learning approach application in predicting atypical metastasis in colorectal cancer after resection. Our study also has several limitations. First, the number of subjects in our study is limited to a single center and is relatively small, suggesting that a multicenter study with a large sample size is needed. Second, the region of interest was manually placed, which may have affected our measurement, although the radiologist was blinded to the clinical information. Third, machine learning models are relatively simple to develop. Recent advances in cutting-edge techniques may enable ensemble learning algorithms to help improve the reliability and robustness of machine learning models.

## Data Availability

The raw data supporting the conclusions of this article will be made available by the authors, without undue reservation.
